# Evaluation of the Effectiveness of a Novel Brain-Computer Interface Neuromodulative Intervention to Relieve Neuropathic Pain Following Spinal Cord Injury: Protocol for a Single-Case Experimental Design With Multiple Baselines

**DOI:** 10.2196/20979

**Published:** 2020-09-29

**Authors:** Negin Hesam-Shariati, Toby Newton-John, Avinash K Singh, Carlos A Tirado Cortes, Tien-Thong Nguyen Do, Ashley Craig, James W Middleton, Mark P Jensen, Zina Trost, Chin-Teng Lin, Sylvia M Gustin

**Affiliations:** 1 Centre for Pain IMPACT, Neuroscience Research Australia Sydney Australia; 2 School of Psychology University of New South Wales Sydney Australia; 3 Graduate School of Health University of Technology Sydney Sydney Australia; 4 School of Computer Science University of Technology Sydney Sydney Australia; 5 John Walsh Centre for Rehabilitation Research, Northern Clinical School University of Sydney Kolling Institute Sydney Australia; 6 Department of Rehabilitation Medicine University of Washington Seattle, WA United States; 7 Department of Physical Medicine and Rehabilitation Virginia Commonwealth University Richmond, VA United States

**Keywords:** EEG neurofeedback, neuropathic pain, spinal cord injury, thalamus, serious games, brain-machine interface, brain-computer interface, single-case experimental design

## Abstract

**Background:**

Neuropathic pain is a debilitating secondary condition for many individuals with spinal cord injury. Spinal cord injury neuropathic pain often is poorly responsive to existing pharmacological and nonpharmacological treatments. A growing body of evidence supports the potential for brain-computer interface systems to reduce spinal cord injury neuropathic pain via electroencephalographic neurofeedback. However, further studies are needed to provide more definitive evidence regarding the effectiveness of this intervention.

**Objective:**

The primary objective of this study is to evaluate the effectiveness of a multiday course of a brain-computer interface neuromodulative intervention in a gaming environment to provide pain relief for individuals with neuropathic pain following spinal cord injury.

**Methods:**

We have developed a novel brain-computer interface-based neuromodulative intervention for spinal cord injury neuropathic pain. Our brain-computer interface neuromodulative treatment includes an interactive gaming interface, and a neuromodulation protocol targeted to suppress theta (4-8 Hz) and high beta (20-30 Hz) frequency powers, and enhance alpha (9-12 Hz) power. We will use a single-case experimental design with multiple baselines to examine the effectiveness of our self-developed brain-computer interface neuromodulative intervention for the treatment of spinal cord injury neuropathic pain. We will recruit 3 participants with spinal cord injury neuropathic pain. Each participant will be randomly allocated to a different baseline phase (ie, 7, 10, or 14 days), which will then be followed by 20 sessions of a 30-minute brain-computer interface neuromodulative intervention over a 4-week period. The visual analog scale assessing average pain intensity will serve as the primary outcome measure. We will also assess pain interference as a secondary outcome domain. Generalization measures will assess quality of life, sleep quality, and anxiety and depressive symptoms, as well as resting-state electroencephalography and thalamic γ-aminobutyric acid concentration.

**Results:**

This study was approved by the Human Research Committees of the University of New South Wales in July 2019 and the University of Technology Sydney in January 2020. We plan to begin the trial in October 2020 and expect to publish the results by the end of 2021.

**Conclusions:**

This clinical trial using single-case experimental design methodology has been designed to evaluate the effectiveness of a novel brain-computer interface neuromodulative treatment for people with neuropathic pain after spinal cord injury. Single-case experimental designs are considered a viable alternative approach to randomized clinical trials to identify evidence-based practices in the field of technology-based health interventions when recruitment of large samples is not feasible.

**Trial Registration:**

Australian New Zealand Clinical Trials Registry (ANZCTR) ACTRN12620000556943; https://bit.ly/2RY1jRx

**International Registered Report Identifier (IRRID):**

PRR1-10.2196/20979

## Introduction

### Background

Approximately 50% of individuals with spinal cord injury (SCI) report ongoing neuropathic pain at or below the level of injury [1-3]. Neuropathic pain is often accompanied by depression, anxiety, and poor sleep quality in this population [4,5], resulting in decreased health-related quality of life [6]. Despite the availability of analgesic medications and other pain therapies, no effective treatment has been found that benefits the majority of individuals with SCI, and most of the available treatments have significant negative side effects or risks of serious adverse events [7,8]. For example, the most powerful analgesics provide about a 50% reduction in pain intensity for only one-third of individuals with SCI [9], and they are associated with severe adverse effects such as toxicity [7]. While nonpharmacological interventions have minimal negative side effects [10-12], a Cochrane systematic review for chronic pain following SCI [8] found limited evidence of pain reduction across trials using repetitive transcranial magnetic stimulation, cranial electrotherapy stimulation, transcutaneous electrical nerve stimulation, acupuncture, hypnosis, or cognitive behavioral therapy. Hence, numerous people with SCI experience ongoing neuropathic pain on a daily basis with no access to effective treatment regimens.

### The Critical Role of the Thalamus in Neuropathic Pain Following SCI

Although many brain regions are involved in the experience of neuropathic pain, Gustin and colleagues have identified the key role of the thalamus in the development and maintenance of neuropathic pain following SCI. They have found that neuropathic pain after SCI is associated with altered thalamic volume [13], neurochemistry [14], and blood flow [14]. Gustin and colleagues [14] suggested that a loss of cortically projecting ventral posterior thalamus neurons results in decreased excitatory input to the thalamic reticular nucleus. As a consequence of decreased thalamic reticular nucleus activity, the γ-aminobutyric acid (GABA) content of the thalamus is reduced. This reduction in thalamic reticular nucleus inhibitory output disrupts normal thalamocortical rhythms. It is postulated that this disruption of thalamocortical rhythms results in ongoing neuropathic pain following SCI [14,15].

The disruption in thalamocortical rhythms (thalamocortical dysrhythmia) can be detected by surface electroencephalography (EEG) [15,16]. EEG signals can be assessed as different frequency bands, such as theta (4-7 Hz), alpha (8-12 Hz), and beta (13-30 Hz). Thalamocortical dysrhythmia is characterized by a common resting-state EEG pattern of increased theta and low-frequency alpha rhythms [17,18]. The increased theta and low alpha frequency powers are further associated with an increase in beta frequency power [19]. For example, Sarnthein and colleagues [15] showed increased EEG activity in delta (2-3.5 Hz), theta (4-7.5 Hz), and beta (13-21 Hz) frequency powers in individuals with neuropathic pain compared with healthy study participants. Boord and colleagues [20], as well as Jensen and colleagues [16], showed similar results for individuals with SCI neuropathic pain having increased theta and decreased alpha frequency powers compared with individuals with SCI who had no pain and healthy study participants.

### Changing Brain Rhythms Reduces Neuropathic Pain Following SCI

There is accumulating evidence that thalamocortical dysrhythmia can be self-regulated by neuromodulative interventions [21]. For example, brain-computer interface (BCI) systems have been used to reduce neuropathic pain after SCI via EEG neurofeedback [22,23]. In BCI-based neuromodulative (BCI-N) interventions, the electrical brain activity is monitored, processed, and provided back to participants in real time via visual or auditory feedback. Using this feedback, individuals can learn to regulate their brain activity in a way that reduces their pain.

Three single-arm trials have demonstrated that BCI-N interventions can reduce SCI neuropathic pain [22-24]. These studies used a BCI-N protocol, which consisted of suppressing theta and low-frequency alpha rhythms (4-8 Hz), and high-frequency beta rhythms (20-30 Hz), along with enhancing high-frequency alpha rhythms (9-12 Hz). Although these preliminary studies suggested that BCI-N can be effective in reducing SCI neuropathic pain, further studies are needed to provide more definitive evidence regarding the effectiveness of BCI-N interventions for people with neuropathic pain following SCI.

Current BCI-N interventions for both SCI neuropathic pain [20,22,24] and chronic pain [25-27] have mostly relied on a single mode of virtual interaction, such as increasing or decreasing the height of a bar presented on a computer screen. However, preliminary evidence suggests that greater pain relief may be achieved from interactive, goal-directed engagement with a gaming environment, in comparison with a single virtual interaction format [28-31]. The increased analgesic effect may be due to the greater cortical involvement, which occurs during engagement with multiple scenarios in a goal-directed manner such as gaming [32]. Based on this evidence, we will use a self-developed BCI-N intervention that uses 3 virtual interactive scenarios in a goal-directed gaming environment to reduce SCI neuropathic pain.

### Objectives

The primary objective is to evaluate the effectiveness of a multiday course of BCI-N intervention in a gaming environment to provide pain relief for individuals with SCI neuropathic pain. The secondary objective is to assess the intervention’s effectiveness on participants’ pain through pain interference. We will also determine whether the BCI-N intervention improves mood, sleep quality, quality of life, and well-being. Lastly, we will explore the neural mechanisms underlying the effect of a BCI-N intervention on SCI neuropathic pain. In particular, we will measure resting-state EEG and levels of thalamic GABA content pre- and postintervention.

## Methods

### Study Design

This study will be conducted based on a single-case experimental design (SCED) with multiple baselines across participants. The SCED is a powerful and effective method that is increasingly used in clinical trial designs [33,34] to evaluate preliminary effectiveness of an intervention [35-37]. Indeed, it has been argued that under the right circumstances, highly controlled SCEDs should be considered on par with traditional group-based randomized clinical trials [38]. An important strength of SCEDs with multiple baselines across participants is that it allows one to determine whether any change observed in the dependent variables occurs when, and only when, the intervention is directed at a particular participant [36]. As a result, the individualized findings of a highly controlled SCED trial can be accumulated to produce results equivalent to those found in randomized clinical trials but requires fewer participants for the same power [38,39]. Thus, the SCED approach is particularly useful when recruiting a large number of individuals into a clinical trial is not feasible.

The SCED method is based on assessing the dependent variables (in this case, pain intensity and pain interference) repeatedly for each of the participants across phases. The design of this study will be AB + follow-ups, where A refers to the baseline phase, B is the intervention phase, and both are followed by a follow-up phase. In addition, we will conduct a further follow-up phase 3 months after completion of the intervention. We will conduct and report the SCED study in accordance with the Single-Case Reporting Guideline in Behavioural Interventions (SCRIBE) 2016 Statement [40]. To meet the SCRIBE reporting standards, there must be at least three participants in a SCED with multiple baselines and more than five assessments of each dependent variable in each phase.

### Participants

We will recruit 3 individuals with complete thoracic SCI (American Spinal Injury Association Impairment Scale A) for this study. Participants need to meet the following inclusion criteria: (1) aged 18 to 80 years, (2) having persistent neuropathic pain for 6 months or longer, (3) having an average pain intensity of 2 or more (out of 10) in the past week on a visual analog scale (VAS; with 0 cm reflecting no pain to 10 cm reflecting the maximum pain imaginable), (4) being medically stable, and (5) demonstrating an ability to use the VAS. With regard to the neuroimaging component of the study, we will exclude individuals who have metal objects inside their body (eg, stents, metal clips, implants, and shrapnel).

### Procedure

**Figure 1:**
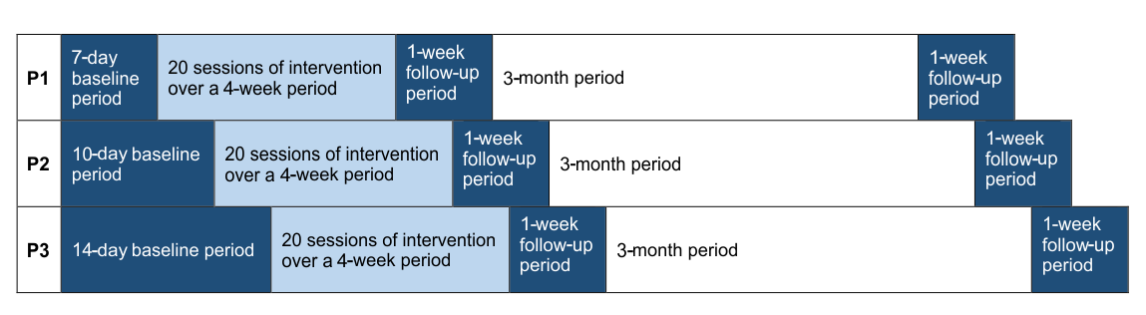
Study procedure. Each participant (P1-3) will be randomly allocated to 1 of 3 baseline periods. Each baseline phase will be followed by 20 days of a brain-computer interface (BCI)-based neuromodulative intervention over a 4-week period, and there will be a 1-week follow-up period for all participants. A further 1-week follow-up will take place 3 months after completion of the intervention.

We will randomly assign 3 participants to different baseline durations of the SCED using a simple randomization method [41]. Conventionally, a minimum of 3 baseline observation days are required to establish stability for the dependent variable [42]; however, more observations are preferred. In this study, we will obtain a stable baseline by using 7 days of observation. We will randomly assign each participant to 1 of 3 experimental conditions characterized by the length of the baseline phase. The first participant will have a 7-day baseline, the second participant will have a 10-day baseline, and the third participant will have a 14-day baseline. All participants will start the baseline phase on the same day. Each baseline phase will be followed by 20 days of a 30-minute BCI-N intervention over a 4-week period. Subsequently, there will be a 1-week follow-up for each participant to continue reporting the primary and secondary outcome measures in order to monitor possible changes in pain intensity and pain interference after completion of the intervention. In addition, we will conduct a further 1-week follow-up 3 months after completion of the intervention. Figure 1 summarizes the study procedure.

### Intervention

**Figure 2:**
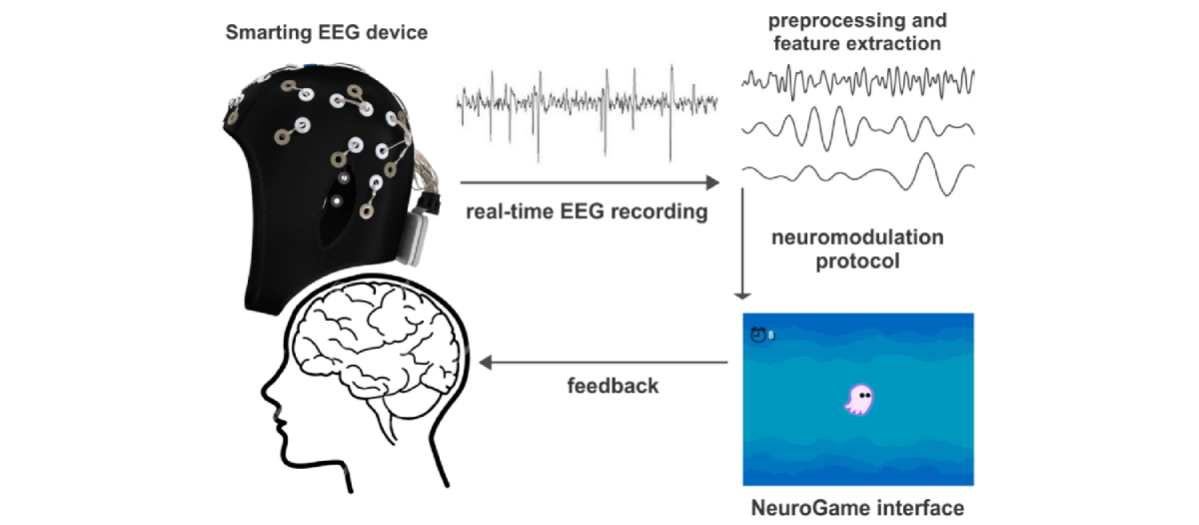
Neurofeedback loop of the brain-computer interface-based neuromodulative intervention (Floating Jellyfish). EEG: electroencephalogram.

Each participant will receive 30-minute daily sessions of the BCI-N intervention for 20 days over a 4-week period in their home. Each session will involve two 15-minute BCI-N interventions divided by a 5-minute break, and each session will start and finish with measurement of the resting-state EEG levels. The BCI-N treatment incorporates an interactive gaming interface (ie, NeuroGame), and a neuromodulation protocol targeted to suppress theta and low alpha (4-8 Hz) and high beta (20-30 Hz) band powers and to enhance high alpha (9-12 Hz) band power (Figure 2). We will acquire the EEG using the EEG system SMARTING device (mBrainTrain; see Figure 2). During the BCI-N intervention, neurofeedback will be provided on SCI neuropathic pain-related regions of the brain, such as C3 and C4 [24]. In particular, the EEG signals will be processed in real time with custom scripts in MATLAB R2020b (MathWorks) using EEGLAB [43] functions. The real-time EEG processing will include extracting the power from the frequencies of interest (ie, 4-8 Hz, 9-12 Hz, and 20-30 Hz) and transferring the information to the NeuroGame interface. The NeuroGame interface was developed using the Unity 3D game engine (Unity Technologies 2019.2.6f1). Our game scenario is based on an online game called A Waffles Fate [44]. We modified the concept from a navigating ghost to a jellyfish. Our scenario, called Floating Jellyfish, provides neurofeedback in an interactive, goal-directed, virtual gaming environment. The visual feedback of the Floating Jellyfish game scenario is as follows: When only 1 EEG frequency band power is suppressed or reinforced as desired, the jellyfish changes color; when 2 frequency band powers are activated correctly, the jellyfish starts to move; and when all 3 band powers are activated, the ocean background changes color, and a seconds timer begins. Points accumulate only for those seconds that the participant keeps all 3 bands activated. The aim of the game is to receive as many points as possible. Points are summed across each 30-minute daily session of BCI-N treatment. Furthermore, the total points scored will be summed across all 20 sessions of BCI-N treatment, with the aim being to accrue as many points in total as possible. The within- and between-session points totals will also provide valuable information about the participants’ experience, engagement, and progression through the game.

### Outcome Measures

#### Primary Outcome Measure

The VAS will serve as the primary outcome measure of SCI neuropathic pain. We will ask the study participants to rate the average intensity of pain during 3 specific epochs each day, using a VAS. The VAS is a 10-cm horizontal line with “No Pain” at one end and “Maximum Pain Imaginable” at the other end. Respondents are asked to make a mark along the line that represents their pain intensity. At 12 o’clock (noon), participants will rate the average intensity of the pain they experienced from the time they woke up that day until noon. At 6 PM, they will rate their average pain intensity between noon and 6 PM. Finally, at the time they go to bed, they will rate the average of the pain intensity they experienced between 6 PM and the time they went to bed. The mathematical average of the 3 ratings will then be computed to represent that participant’s average daily pain intensity. If any ratings are missing, the score will be the average of the ratings obtained. Although consensus groups recommend the numeric rating scale over the VAS in pain clinical trials because some individuals have problems with understanding the VAS [45], we chose the VAS because our experience with the SCI population is that they prefer it over the numeric rating scale. Indeed, a great deal of evidence supports the reliability and validity of the VAS for assessing pain intensity among individuals who are able to use this measure [46]. Participants will complete this daily paper-and-pencil pain diary during all 3 phases; that is, the baseline (7, 10, or 14 days), intervention (20 days), and follow-up (7 days) phases.

#### Secondary Outcome Measure

Pain interference will serve as a secondary outcome measure. We will assess the degree of pain interference by 6 items from the Brief Pain Inventory (BPI) [47]. These items will assess general activity, normal work, relations with other people, enjoyment of life, mood, and sleep on a scale of 0 to 10 (0 = “does not interfere” and 10 = “completely interferes”). Degree of pain interference will be assessed daily in the evenings during the baseline, intervention, and follow-up phases.

We will have daily contact with the participants, both to address any questions they may have about the VAS and BPI measures, and to ensure they are completing their pain diaries and BPIs during the times they are supposed to.

#### Generalization Measures

We will collect generalization measures to evaluate whether the effect of the treatment extends beyond improvements in the primary and secondary outcomes [37]. For example, in this study, measures will be administered to assess whether any treatment effects generalize to behaviors and outcomes other than the pain intensity and pain interference. The generalization measures are important to strengthen the external validity of the research findings [37]. In this study, these measures will include resting-state EEG, neuroimaging data (in this case, thalamic GABA content), and psychological questionnaires measuring levels of anxiety and depression, quality of life, well-being, and sleep quality.

We will administer the psychological questionnaires at 5 time points: (1) prior to the baseline phase, (2) on the last day of the baseline phase, (3) on the last day of the intervention phase, (4) on the last day of the first follow-up phase, and (5) on the last day of the second follow-up phase. We will collect the resting-state EEG and the neuroimaging data at 2 time points: prior to and following completion of the intervention.

#### Psychological Questionnaires

Participants will complete the 36-item Short Form Health Survey modified for SCI (SF-36 walk-wheel) [48], the COMPAS-W Scale of Wellbeing [49], the State Anxiety Inventory (SAI) [50], the Beck Depression Inventory (BDI) [51], and the Medical Outcomes Study Sleep Scale (MOS-SS) [52]. These questionnaires are self-report measures. The SF-36 [48] is a frequently used instrument that measures global health-related quality of life. The SF-36 [48] consists of 8 scales: physical functioning, physical role functioning, bodily pain, general health perceptions, vitality, social role functioning, emotional role functioning, and mental health. Higher scores represent a better health status. The 26 items of the COMPAS-W [49] scale measures both subjective and psychological well-being. It consists of 6 subscales, which provide an indication for different aspects of well-being: composure, own worth, mastery, positivity, achievement, and satisfaction. Higher scores represent higher levels of well-being. The 20-item SAI [50] assesses state anxiety, with higher scores indicating greater anxiety. The 21-item BDI [51] is used to assess the severity of depressive symptoms, with a higher score indicating a greater number of depressive symptoms. The 12-item MOS-SS [52] assesses 6 key factors of sleep: sleep initiation, maintenance, respiratory problems, quantity, perceived adequacy, and somnolence. Higher scores reflect more sleep quality and quantity. Lastly, we will use the Neuropathic Pain Scale (NPS) [53] to evaluate the specific qualities of SCI neuropathic pain, such as sharp, hot, dull, cold, and sensitive.

#### Resting-State EEG Measures

We will record resting-state EEG with the participants’ eyes closed (3 minutes) and eyes open (3 minutes) using the 24-channel EEG device SMARTING. The electrode placements are according to the standard 10-20 locations. The electrode impedance will be kept under 5 kΩ, and the sampling frequency will be 500 Hz.

#### Neuroimaging Measures

Participants will lie supine, headfirst, on the bed of a 3-T magnetic resonance imaging machine (Ingenia; Philips) with their head immobilized in a 32-channel head coil. We will use multiplanar (axial, sagittal, coronal) reformats for voxel placement. GABA-edited Meshcher-Garwood Point Resolved Spectroscopy (MEGA-PRESS) [54] will be acquired from a voxel (2×2×2 cm^3^), which will be centered in the contralateral (to pain) thalamus in each participant.

#### Safety, Feasibility, and Experience Measures

We will collect participants’ feedback regarding perceived safety and feasibility of the intervention following each session of BCI-N treatment using study-specific questions (qualitative data). Following the completion of 20 sessions of BCI-N treatment, the participants will complete the Usefulness, Satisfaction, and Ease of Use [55] questionnaire and the Patient Global Impression of Change [56] scale to assess the feasibility and perceived change in pain, respectively. Additionally, we will conduct an unstructured interview after completion of the intervention to assess each participant’s experience with the BCI-N system and the mental strategy or strategies they used during neurofeedback. The topics that will be addressed will include ease of use, barriers to utilization, participant burden, technology acquisition, and individualized mental strategy. We will use the information gathered from the unstructured interviews to optimize the technological design, clinical protocols, and assessment procedures for subsequent larger trials.

### Data Analysis

#### Primary and Secondary Outcomes Analysis

We will analyze primary and secondary outcomes separately based on the SCED analysis. The SCED analysis mainly relies on visual inspection. However, we will inspect and analyze the outcome measures from this study using both visual analysis and supplementary statistical analysis [57]. In the visual analysis, the baseline phase establishes a benchmark against the intervention phase to investigate any natural change in the outcome of interest. We will use a structured analysis to investigate whether the treatment-induced changes in the primary and secondary outcomes are reliable and consistent across participants [57,58].

The data across all phases will be scattered and visually analyzed using both within-phase and between-phase analyses [35,59]. Within-phase analysis refers to evaluating the data patterns in each phase, and between-phase analysis evaluates the data overlap between phases and the data pattern consistency across participants. To decide whether and to what extent the intervention has had an effect on the primary and secondary outcome measures, multiple factors need to be considered in interpreting the data: (1) trend, (2) level, (3) stability, (4) consistency, and (5) overlap.

*Trend* refers to the slope of changes across observations (over time), which we will estimate using the split-middle method [59]. This method is robust to the effects of the autocorrelation of the data. Autocorrelation is the similarity between observations as a function of time, which we will estimate using the delta-recursive method [60].

*Level* refers to the rate of change either within a phase or from one phase to the next phase [59]. The between-phase relative level change is the proportional change from the last half of the baseline to the first half of the intervention phase, whereas the absolute level change is the immediacy of change from the last session of the baseline to the first session of the intervention using median values.

*Stability* refers to the percentage of data points on or within a stability envelope. We will evaluate the stability envelope of the baseline phase by a criterion of 80% of data points being within 25% of the median value [59]. A requirement for demonstrating an effect of the intervention is the stability of the baseline phase compared with the intervention phase.

*Consistency* refers to the extent that data patterns of the same phase are similar across participants (eg, consistency of the baseline phase between participants). We will evaluate this by the consistency of data patterns approach. In addition, we will apply the consistency of the effects to assess the replication of the between-phase change across participants [61].

*Overlap* is the percentage of data points from the intervention phase that overlap with the data from the baseline phase. The higher percentage of nonoverlap data determines a larger intervention effect. Nonoverlap indices are more robust than indices of mean or median level changes across phases. Nonoverlap methods do not rely on means or medians, but rather consider the individual values of all data points in pairwise comparisons across phases [62]. These methods are distribution free, meaning that they do not require parametric assumptions.

One of the most robust nonoverlap methods is the Tau effect size [63,64], which is based on the pairwise comparison. Pairwise comparison between 2 measures is determined to be concordant (positive), discordant (negative), or tied [63,65]. In a concordant pair, there is an increase between 2 measures; in a discordant pair, there is a decrease; and in a tied pair, both measures are equal. Tau is calculated using the formula 
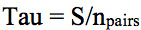
, where S is the Kendall rank correlation score, calculated as n_c_ – n_d_ (number of discordant pairs subtracted from the number of the concordant pairs), and n_pairs_ is the number of all pairs.

Tau-U effect size [63,65] extends Tau in an attempt to control for the undesirable trend in the baseline phase by estimating and removing it. A SCED with AB design consists of both within-phase (A-to-A, B-to-B) and between-phase (A-to-B) pairwise comparisons. A-to-A and B-to-B comparisons characterize the trend within phase A and phase B, respectively, and A-to-B comparisons describe the difference between phase A and phase B. To remove the within-phase trend from the between-phase difference, we will use the following formula: 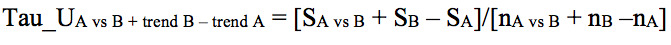 We will use Tau-U to provide an overall treatment effect size across the 3 participants.

#### Generalization Measures Analysis

##### Psychological Questionnaires

We will calculate and extract total scores and subscores of the psychological questionnaires (SF-36 walk-wheel [48], COMPAS-W [49] SAI [50], BDI [51], MOS-SS [66], and NPS [52]) for further analysis (see the Reliable Change Index subsection below).

##### Resting-State EEG

We will analyze resting-state EEG data using custom scripts based on the EEGLAB toolbox [43] functions in MATLAB. We will apply a band-pass filter (1-30 Hz) to the EEG data after downsampling to 250 Hz. Then, the EEG data will be rereferenced to the average of all 24 electrodes. We will use an artifact subspace reconstruction [67] method with default parameters for denoising and removing movement artifacts. Subsequently, we will use power spectral density to estimate the intended frequency band powers (ie, theta, alpha, and high beta) from the cleaned data. Finally, we will extract the frequency band power values for further analysis (see Reliable Change Index below).

##### Neuroimaging

We will analyze the acquired spectra using the Java-based magnetic resonance user interface (jMRUI version 6.0; MRUI Consortium). First, we will remove the dominant water resonance using the Hankel Lanczos singular valve decomposition algorithm. The ON and OFF spectral subsets will be summed, producing single ON and OFF 68-ms subspectra for each spectra dataset. These 68-ms subspectra will then be subtracted, resulting in GABA-edited difference spectra to measure GABA concentration at 3.01 ppm. We will quantify GABA using AMARES, a nonlinear least-squares fitting algorithm operating in the time domain. Peak fitting for GABA will be performed after manually defining the center frequency and line width of the GABA peak and modelling the GABA peak as a singlet. We will use Lorentzian curves to obtain the peak amplitude for this resonance. The OFF spectral subsets will be summed, producing single OFF 68-ms subspectra for each spectra dataset to measure creatine concentration at 3.02 ppm. We will then phase the single OFF 68-ms subspectra with respect to both the zero- and first-order phase. Spectral fitting in AMARES will be performed after manually defining the center frequency and line width of the creatine peak and modelling the creatine peak as a singlet. We will use Gaussian curves to obtain the peak amplitude for this resonance. Lastly, the GABA to creatine ratios will be calculated and extracted for further analysis (see Reliable Change Index below).

##### Reliable Change Index

We will evaluate changes in generalization measures such as power spectral density, GABA to creatine ratios, and total scores and subscores of the psychological questionnaires using the Reliable Change Index (RCI) [68,69]. We will use the RCI to evaluate the reliability of change over time for individual data. This index indicates whether the change score between 2 time points (eg, pre- and postintervention) for the same individual is considered clinically significant.

RCI is a ratio of the actual observed difference by the standard error of the difference (SE_diff_): RCI = [M_post_ – M_pre_]/SE_diff_, and SE_diff_ = SD√2(1 – r), where SD is the standard deviation of the measurement and r is the reliability coefficient of the measure.

## Results

This clinical trial has been approved by the University of New South Wales Human Research Ethics Committee (approval number: HC190411) and the University of Technology Sydney Human Ethics Committee (approval number: ETH19-4090). Additionally, this study is registered through the Australian and New Zealand Clinical Trials Registry (registration number: ACTRN12620000556943). We plan to commence the trial in October 2020 and expect to publish the results by the end of 2021.

## Discussion

### Overview

Preliminary data in support of BCI-N treatment for SCI neuropathic pain have been reported [24,26]. However, further studies are needed to provide more definitive evidence of the effectiveness of this intervention. This clinical trial using SCED methodology has been designed to evaluate the effectiveness of a novel BCI-N treatment for people with neuropathic pain after SCI.

The BCI-N system of this study will address a key limitation of previous EEG neurofeedback interventions, which have mostly relied on a single form of virtual interaction [20,22,24]. Studies have shown greater cortical involvement [32] and pain reduction [28-31] during the interactive engagement with multiple gaming scenarios than with 1 form of virtual interaction. Based on this evidence, we will use a self-developed BCI-N treatment, which consists of 3 interactive virtual scenarios in a goal-directed gaming environment. Further, the BCI-N system used in this study can provide neurofeedback simultaneously on different brain regions, and hence can be individualized to each participant’s needs. For example, for a participant with bilateral pain, both C3 and C4 EEG channels can be targeted, whereas for a participant with unilateral pain, only C3 or C4 will be targeted during the BCI-N intervention. Additionally, the reinforcement and suppression criteria (ie, the baseline thresholds) for the targeted frequency bands can be customized according to the participant’s progress during the intervention. For example, we can change the difficulty of the game by modifying the baseline thresholds as a way to increase motivation.

SCED is a powerful design to establish guidelines for evidence-based interventions [35]. The key to the SCED method with multiple baselines is that the intervention is introduced in a staggered sequence [35], and the outcome of interest, that is, average pain intensity, is measured repeatedly during different phases. The staggered introduction of the intervention allows for drawing conclusions regarding intervention effects distinct from those associated with maturation, experience, learning, or practice [70]. The SCED methodology accounts for potential confounders, such as environment factors, which may affect primary and secondary outcome measures. Unlike traditional group designs, a SCED can address the effects of the intervention at the individual’s level in a controlled and unbiased way by randomly allocating different baseline periods across participants.

Investigating our novel self-developed BCI-N intervention with a SCED and evaluating it with visual and statistical analyses will provide a rigorous methodology for this study. The high internal validity of a well-implemented SCED study allows for the results of the data analyses to draw reliable conclusions about the effectiveness of the intervention [42,71]. Experimental control is established when the effects of the intervention are repeatedly and reliably demonstrated within a single participant or across a small number of participants. In the multiple-baseline design, each participant will be their own control and will provide an instance of the intervention’s effect replication. This within-study replication is the basis of internal validity in SCEDs. By replicating an investigation across different participants, the generalization of the intervention effects can be examined and hence potentially increase the external validity [35]. Additionally, SCEDs are ideal for researchers working with small or very heterogeneous populations in the development and implementation phases of novel research studies. Thus, the SCED with multiple baselines is considered a viable alternative approach to randomized clinical trials for demonstrating the effectiveness of an intervention when the recruitment of large samples is not feasible. [42].

### Limitations

The SCED trial with multiple baselines will not be able to demonstrate effectiveness of the BCI-N treatment if 1 of the 3 participants drops out during the baseline or intervention phases. To address this limitation, we will perform a mock BCI-N treatment session for each participant prior to commencement of the trial in order to increase compliance and ensure a high comfort level of the EEG headset, a comprehensive understanding of the treatment protocol, assessment procedures, and performance of gameplay during the neurofeedback session.

## Abbreviations

BCI: brain-computer interface
BCI-N: BCI-based neuromodulative
BDI: Beck Depression Inventory
BPI: Brief Pain Inventory
EEG: electroencephalography
GABA: γ-aminobutyric acid
MEGA-PRESS: Meshcher-Garwood Point Resolved Spectroscopy
NPS: Neuropathic Pain Scale
RCI: Reliable Change Index
SAI: State Anxiety Inventory
SCED: single-case experimental design
SCI: spinal cord injury
SCRIBE: Single-Case Reporting Guideline in Behavioural Interventions
SF-36: 36-item Short Form Health Survey
VAS: visual analog scale

## References

[1] Siddall PJ, McClelland JM, Rutkowski SB, Cousins MJ (2003). A longitudinal study of the prevalence and characteristics of pain in the first 5 years following spinal cord injury. Pain.

[2] Celik EC, Erhan B, Lakse E (2012). The clinical characteristics of neuropathic pain in patients with spinal cord injury. Spinal Cord.

[3] Gustin SM, Peck CC, Wilcox SL, Nash PG, Murray GM, Henderson LA (2011). Different pain, different brain: thalamic anatomy in neuropathic and non-neuropathic chronic pain syndromes. J Neurosci.

[4] Nicholson B, Verma S (2004). Comorbidities in chronic neuropathic pain. Pain Med.

[5] Tunks ER, Crook J, Weir R (2008). Epidemiology of chronic pain with psychological comorbidity: prevalence, risk, course, and prognosis. Can J Psychiatry.

[6] Jensen MP, Chodroff MJ, Dworkin RH (2007). The impact of neuropathic pain on health-related quality of life: review and implications. Neurology.

[7] Finnerup NB, Attal N, Haroutounian S, McNicol E, Baron R, Dworkin RH, Gilron I, Haanpää M, Hansson P, Jensen TS, Kamerman PR, Lund K, Moore A, Raja SN, Rice ASC, Rowbotham M, Sena E, Siddall P, Smith BH, Wallace M (2015). Pharmacotherapy for neuropathic pain in adults: a systematic review and meta-analysis. Lancet Neurol.

[8] Boldt I, Eriks-Hoogland I, Brinkhof MWG, de Bie R, Joggi D, von Elm E (2014). Non-pharmacological interventions for chronic pain in people with spinal cord injury. Cochrane Database Syst Rev.

[9] Siddall PJ (2009). Management of neuropathic pain following spinal cord injury: now and in the future. Spinal Cord.

[10] Soler MD, Kumru H, Pelayo R, Vidal J, Tormos JM, Fregni F, Navarro X, Pascual-Leone A (2010). Effectiveness of transcranial direct current stimulation and visual illusion on neuropathic pain in spinal cord injury. Brain.

[11] Capel ID, Dorrell HM, Spencer EP, Davis MWL (2003). The amelioration of the suffering associated with spinal cord injury with subperception transcranial electrical stimulation. Spinal Cord.

[12] Tan G, Rintala DH, Jensen MP, Richards JS, Holmes SA, Parachuri R, Lashgari-Saegh S, Price LR (2011). Efficacy of cranial electrotherapy stimulation for neuropathic pain following spinal cord injury: a multi-site randomized controlled trial with a secondary 6-month open-label phase. J Spinal Cord Med.

[13] Gustin SM, Wrigley PJ, Siddall PJ, Henderson LA (2010). Brain anatomy changes associated with persistent neuropathic pain following spinal cord injury. Cereb Cortex.

[14] Gustin SM, Wrigley PJ, Youssef AM, McIndoe L, Wilcox SL, Rae CD, Edden RAE, Siddall PJ, Henderson LA (2014). Thalamic activity and biochemical changes in individuals with neuropathic pain after spinal cord injury. Pain.

[15] Sarnthein J, Stern J, Aufenberg C, Rousson V, Jeanmonod D (2006). Increased EEG power and slowed dominant frequency in patients with neurogenic pain. Brain.

[16] Jensen MP, Sherlin LH, Gertz KJ, Braden AL, Kupper AE, Gianas A, Howe JD, Hakimian S (2013). Brain EEG activity correlates of chronic pain in persons with spinal cord injury: clinical implications. Spinal Cord.

[17] Llinás RR, Ribary U, Jeanmonod D, Kronberg E, Mitra PP (1999). Thalamocortical dysrhythmia: a neurological and neuropsychiatric syndrome characterized by magnetoencephalography. Proc Natl Acad Sci U S A.

[18] Llinás R, Urbano FJ, Leznik E, Ramírez RR, van Marle HJF (2005). Rhythmic and dysrhythmic thalamocortical dynamics: GABA systems and the edge effect. Trends Neurosci.

[19] Vanneste S, Song J, De Ridder D (2018). Thalamocortical dysrhythmia detected by machine learning. Nat Commun.

[20] Boord P, Siddall PJ, Tran Y, Herbert D, Middleton J, Craig A (2008). Electroencephalographic slowing and reduced reactivity in neuropathic pain following spinal cord injury. Spinal Cord.

[21] Jensen MP, Day MA, Miró J (2014). Neuromodulatory treatments for chronic pain: efficacy and mechanisms. Nat Rev Neurol.

[22] Jensen MP, Gertz KJ, Kupper AE, Braden AL, Howe JD, Hakimian S, Sherlin LH (2013). Steps toward developing an EEG biofeedback treatment for chronic pain. Appl Psychophysiol Biofeedback.

[23] Vučković A, Altaleb MKH, Fraser M, McGeady C, Purcell M (2019). EEG correlates of self-managed neurofeedback treatment of central neuropathic pain in chronic spinal cord injury. Front Neurosci.

[24] Hassan MA, Fraser M, Conway BA, Allan DB, Vuckovic A (2015). The mechanism of neurofeedback training for treatment of central neuropathic pain in paraplegia: a pilot study. BMC Neurol.

[25] Caro XJ, Winter EF (2011). EEG biofeedback treatment improves certain attention and somatic symptoms in fibromyalgia: a pilot study. Appl Psychophysiol Biofeedback.

[26] Kayiran S, Dursun E, Dursun N, Ermutlu N, Karamürsel S (2010). Neurofeedback intervention in fibromyalgia syndrome; a randomized, controlled, rater blind clinical trial. Appl Psychophysiol Biofeedback.

[27] Prinsloo S, Gabel S, Lyle R, Cohen L (2014). Neuromodulation of cancer pain. Integr Cancer Ther.

[28] Hoffman HG, Sharar SR, Coda B, Everett JJ, Ciol M, Richards T, Patterson DR (2004). Manipulating presence influences the magnitude of virtual reality analgesia. Pain.

[29] Gordon NS, Merchant J, Zanbaka C, Hodges LF, Goolkasian P (2011). Interactive gaming reduces experimental pain with or without a head mounted display. Comput Hum Behav.

[30] Zavarize SF, Paschoal MA, Wechsler SM (2016). Efects of physiotherapy associated to virtual games in pain perception and heart rate variability in cases of low back pain. Manual Ther Posturol Rehabil J.

[31] Fairclough SH, Stamp K, Dobbins C, Poole HM (2020). Computer games as distraction from PAIN: effects of hardware and difficulty on pain tolerance and subjective IMMERSION. Int J Hum Comput Stud.

[32] Bakaoukas AG, Coada F, Liarokapis F (2016). Examining brain activity while playing computer games. J Multimodal User Interfaces.

[33] Purswell KE, Ray DC (2014). Research with small samples: considerations for single case and randomized small group experimental designs. Couns Outcome Res Eval.

[34] Kratochwill TR, Hitchcock JH, Horner RH, Levin JR, Odom SL, Rindskopf DM, Shadish WR (2013). Single-case intervention research design standards. Remedial Spec Educ.

[35] Byiers BJ, Reichle J, Symons FJ (2012). Single-subject experimental design for evidence-based practice. Am J Speech Lang Pathol.

[36] Dallery J, Cassidy RN, Raiff BR (2013). Single-case experimental designs to evaluate novel technology-based health interventions. J Med Internet Res.

[37] Krasny-Pacini A, Evans J (2018). Single-case experimental designs to assess intervention effectiveness in rehabilitation: a practical guide. Ann Phys Rehabil Med.

[38] Romeiser Logan L, Hickman RR, Harris SR, Heriza CB (2008). Single-subject research design: recommendations for levels of evidence and quality rating. Dev Med Child Neurol.

[39] Shamseer L, Sampson M, Bukutu C, Schmid CH, Nikles J, Tate R, Johnston BC, Zucker D, Shadish WR, Kravitz R, Guyatt G, Altman DG, Moher D, Vohra S, CENT Group (2015). CONSORT extension for reporting N-of-1 trials (CENT) 2015: explanation and elaboration. BMJ.

[40] Tate RL, Perdices M, Rosenkoetter U, Shadish W, Vohra S, Barlow DH, Horner R, Kazdin A, Kratochwill T, McDonald S, Sampson M, Shamseer L, Togher L, Albin R, Backman C, Douglas J, Evans JJ, Gast D, Manolov R, Mitchell G, Nickels L, Nikles J, Ownsworth T, Rose M, Schmid CH, Wilson B (2017). The single-case reporting guideline in behavioural interventions (SCRIBE) 2016 statement. Neuropsychol Rehabil.

[41] Altman DG, Bland JM (1999). How to randomise. BMJ.

[42] Kazdin AE (2011). Single-Case Research Designs: Methods for Clinical and Applied Settings. 2nd edition.

[43] Delorme A, Makeig S (2004). EEGLAB: an open source toolbox for analysis of single-trial EEG dynamics including independent component analysis. J Neurosci Methods.

[44] Calice N (2020). A Waffles Fate. itch.io.

[45] Dworkin RH, Turk DC, Farrar JT, Haythornthwaite JA, Jensen MP, Katz NP, Kerns RD, Stucki G, Allen RR, Bellamy N, Carr DB, Chandler J, Cowan P, Dionne R, Galer BS, Hertz S, Jadad AR, Kramer LD, Manning DC, Martin S, McCormick CG, McDermott MP, McGrath P, Quessy S, Rappaport BA, Robbins W, Robinson JP, Rothman M, Royal MA, Simon L, Stauffer JW, Stein W, Tollett J, Wernicke J, Witter J, IMMPACT (2005). Core outcome measures for chronic pain clinical trials: IMMPACT recommendations. Pain.

[46] Hawker GA, Mian S, Kendzerska T, French M (2011). Measures of adult pain: visual analog scale for pain (VAS pain), numeric rating scale for pain (NRS pain), McGill pain questionnaire (MPQ), short-form McGill pain questionnaire (SF-MPQ), chronic pain grade scale (CPGS), short form-36 bodily pain scale (SF-36 BPS), and measure of intermittent and constant osteoarthritis pain (ICOAP). Arthritis Care Res (Hoboken).

[47] Cleeland CS, Turk DC, Melzack R (1992). The brief pain inventory. Handbook of Pain Assessment.

[48] Lee BB, Simpson JM, King MT, Haran MJ, Marial O (2009). The SF-36 walk-wheel: a simple modification of the SF-36 physical domain improves its responsiveness for measuring health status change in spinal cord injury. Spinal Cord.

[49] Gatt JM, Burton KLO, Schofield PR, Bryant RA, Williams LM (2014). The heritability of mental health and wellbeing defined using COMPAS-W, a new composite measure of wellbeing. Psychiatry Res.

[50] Spielberger CD, Gorsuch RL, Lushene R (1970). Manual for the State-Trait Anxiety Inventory.

[51] Beck AT, Ward CH, Mendelson M, Mock J, Erbaugh J (1961). An inventory for measuring depression. Arch Gen Psychiatry.

[52] Hays RD, Stewart A, Stewart AL, Ware JE, Jr (1992). Chapter 14. Sleep measures. Measuring Functioning and Well-Being: The Medical Outcomes Study Approach.

[53] Galer BS, Jensen MP (1997). Development and preliminary validation of a pain measure specific to neuropathic pain: the neuropathic pain scale. Neurology.

[54] Edden RAE, Barker PB (2007). Spatial effects in the detection of gamma-aminobutyric acid: improved sensitivity at high fields using inner volume saturation. Magn Reson Med.

[55] Lund AM (2001). Measuring usability with the USE questionnaire. Usability Interface.

[56] Widerström-Noga E, Biering-Sørensen F, Bryce TN, Cardenas DD, Finnerup NB, Jensen MP, Richards JS, Siddall PJ (2014). The international spinal cord injury pain basic data set (version 2.0). Spinal Cord.

[57] Clanchy KM, Tweedy SM, Tate RL, Sterling M, Day MA, Nikles J, Ritchie C (2019). Evaluation of a novel intervention to improve physical activity for adults with whiplash associated disorders: protocol for a multiple-baseline, single case experimental study. Contemp Clin Trials Commun.

[58] Lieberman RG, Yoder PJ, Reichow B, Wolery M (2010). Visual analysis of multiple baseline across participants graphs when change is delayed. School Psychol Q.

[59] Lane JD, Gast DL (2014). Visual analysis in single case experimental design studies: brief review and guidelines. Neuropsychol Rehabil.

[60] Solanas A, Manolov R, Sierra V (2010). Lag-one autocorrelation in short series: estimation and hypotheses testing. Psicológica.

[61] Tanious R, De TK, Michiels B, Van den Noortgate W, Onghena P (2020). Assessing consistency in single-case A-B-A-B phase designs. Behav Modif.

[62] Parker RI, Vannest K (2009). An improved effect size for single-case research: nonoverlap of all pairs. Behav Ther.

[63] Parker RI, Vannest KJ, Davis JL, Sauber SB (2011). Combining nonoverlap and trend for single-case research: Tau-U. Behav Ther.

[64] Parker RI, Vannest KJ, Davis JL (2011). Effect size in single-case research: a review of nine nonoverlap techniques. Behav Modif.

[65] Brossart DF, Laird VC, Armstrong TW (2018). Interpreting Kendall’s Tau and Tau-U for single-case experimental designs. Cogent Psychol.

[66] Stewart AL, Hays RD, Ware JE (1988). The MOS short-form general health survey: reliability and validity in a patient population. Med Care.

[67] Mullen TR, Kothe CAE, Chi YM, Ojeda A, Kerth T, Makeig S, Jung T, Cauwenberghs G (2015). Real-time neuroimaging and cognitive monitoring using wearable dry EEG. IEEE Trans Biomed Eng.

[68] Duff K (2012). Evidence-based indicators of neuropsychological change in the individual patient: relevant concepts and methods. Arch Clin Neuropsychol.

[69] Maassen GH, Bossema E, Brand N (2009). Reliable change and practice effects: outcomes of various indices compared. J Clin Exp Neuropsychol.

[70] Lobo MA, Moeyaert M, Baraldi Cunha A, Babik I (2017). Single-case design, analysis, and quality assessment for intervention research. J Neurol Phys Ther.

[71] Horner RH, Carr EG, Halle J, McGee G, Odom S, Wolery M (2005). The use of single-subject research to identify evidence-based practice in special education. Exceptional Child.

